# Behavioral Changes of Solitary Housed Female Pygmy Slow Lorises (*Nycticebus pygmeaus*) after Introduction into Group Enclosures

**DOI:** 10.3390/ani11092751

**Published:** 2021-09-20

**Authors:** Josue Alejandro, Yumi Yamanashi, Kei Nemoto, Fred B. Bercovitch, Michael A. Huffman

**Affiliations:** 1 Primate Research Institute (PRI), Kyoto University, Inuyama 484-8506, Japan; huffman.michael.8n@kyoto-u.ac.jp; 2 Wildlife Research Center (WRC), Kyoto University, Kyoto 606-8203, Japan; yumi.yamanashi.kycz@gmail.com (Y.Y.); fbercovitch@gmail.com (F.B.B.); 3 Center for Research and Education of Wildlife (CREW), Kyoto City Zoo, Kyoto 606-8333, Japan; 4 Japan Monkey Centre (JMC), Inuyama 484-0081, Japan; kei.nemoto@j-monkey.jp

**Keywords:** pygmy slow loris, animal welfare, captivity, sociality, *Nycticebus pygmeaus*

## Abstract

**Simple Summary:**

Most pygmy slow lorises confiscated in the illegal pet trade cannot be released back into the wild and end up in captivity for the rest of their lives. In the wild, their home ranges are large relative to their body size and they maintain a fairly solitary lifestyle compared to other primates. With the aim of improving their captive wellbeing, we tested to see whether female–female group pairings were in the animals’ best interest by looking at their behaviors before and after being moved to social housing in a larger enriched enclosure. With one nesting site per animal and ample space to avoid each other, all females opted to nest in pairs, not alone, and spent their time socially engaged in affiliative behaviors. We found that female pygmy slow lorises are more social than often assumed and that housing them with conspecifics is probably beneficial to their well-being in captivity.

**Abstract:**

Pygmy slow lorises (*Nycticebus pygmaeus*) are threatened with extinction in the wild. Their nocturnal lifestyle and small size make them difficult to study in their natural habitat, but increasing evidence suggests that they are more social than previously thought. Our study was designed to assess the sociability of pygmy slow lorises by transferring six adult females from solo cages into environmentally enriched group home cages at the Japan Monkey Centre’s Slow Loris Conservation Centre. Two females were paired to create one group, while the other four were placed together in a second group. We compared their social interactions, activity budgets, and postural behaviors before and after social housing was initiated. We found that all-female slow loris groups had a high degree of sociality, preferred to stay close to each other, nested together every night, and spent less time in locomotion and more time grooming than when living alone. These results suggest that female pygmy slow lorises actively seek companions when available. The captive housing of all-female groups of lorises could lead to better husbandry practices and improved animal welfare by allowing them to have conspecific companions. We conclude that isosexual groups of pygmy slow lorises should be preferred over single housing when possible.

## 1. Introduction

Slow lorises (*Nycticebus* spp.) are a taxon of nine nocturnal species that live in Southeast Asia [1,2]. Although lorises have been categorized as ”Endangered” on the IUCN Red List [3], and are included in Appendix I of CITES, a listing that prohibits international trade in specimens, Japan has been a major destination for illegal pet lorises [4]. The illegal smuggling of lorises into Japan, and their rescue by authorities, poses a challenge to animal welfare advocates seeking to provide the best environment possible for them. To improve the conditions of animals found in Japan, recent studies on the behavioral management strategies for male pygmy slow lorises in captivity have reported that social grooming and affiliative contact are important in their social interactions and appear to improve their well-being [5].

In previous studies, pygmy slow lorises have been considered to be among the most solitary of the nocturnal primates [6,7,8]. However, recent studies of both wild and captive populations have reported that they are more social than initially thought [5,9,10]. Due to the difficulty of observing and following nocturnal prosimians, details of their social system and the degree of sociality have been inferred by home range size and overlap [11,12]. Some of these species were thought to maintain contact only during the mating season and between mother and offspring, with such interactions accounting for only about 2% of their entire activity budget [13,14]. Recent radio-tracking studies of Malay slow lorises (*Nycticebus coucang coucang*) in the wild have shown that they form social networks with adults involved in affiliative interactions for up to 8% of their activity budgets [15]. Animals have been seen allogrooming, playing, and traveling together, as well as forming sleeping associations of two to three adults. In Sri Lanka, the closely related slender lorises (*Loris tardigradus*) spent 44% of their nightly activity together with conspecifics, and up to nine individuals occupied the same nest [14]. Captive studies have also noted some degree of sociality in the slender loris [16]. In pygmy slow lorises, interactions arising from home range overlap have been shown to account for up to 5% of their social interactions in the wild [17]. A recent re-evaluation of the evolution of social organization in Lorisiformes has shown that pygmy slow loris have the largest home range size with a multi-female type of mating system [18]. These findings emphasize the need for improvements to captive space in order to promote their natural tendency to have social interactions, and calls for the reconsideration of social management routines for pygmy slow lorises in captivity, as most pygmy slow lorises are still housed alone [19].

Most literature regarding the social management of captive primates has focused on monkeys and apes, with less information reported on the strepsirrhines. They are found in captivity mainly in research facilities, followed by captive breeding colonies, and zoos, with the Duke Lemur Center (DLC) conducting close to 95% of all noninvasive research [20]. Strepsirrhines have been considered an important suborder in understanding primate evolution, as they are considered basal primates [21], yet except for their diurnal lemurs, much of their captive conditions are under-reported. This suborder of primates exhibits a unique set of behaviors such as torpor found in tropical environments seen in bush babies in Africa, lorises of Asia, and lemurs of Madagascar [22]. Many are nectar, exudates, and pollen feeders, providing good models for angio-sperm co-evolution [23]. Their small size and low basal oxygen consumption also provide insights into life history and energy expenditure studies [24]. These factors that make strepsirrhines a unique group within primates also pose challenges in captivity, and what we know of them in the wild might be different and difficult to replicate in captivity. Black lemurs (*Eulemur macaco*) are mainly found in pairs in the wild, and their seasonal food scarcity leads to high food competition for which single or pair living might seem more suitable in captivity to prevent aggression, but when housed in pairs in captivity, they exhibit very low reproductive rates [25]. However, with the addition of nonreproductive males to the groups, reproductive rates increased by 100% [26]. Early pilot studies [27] housing wild caught nocturnal greater galagos (*Galago crassicaudatus*) in groups of 4 (1 male and 3 females) in indoor (12′ × 14′) enclosures found high rates of aggression, with females being removed from the group due to injuries. Animals spent most of their time ignoring and avoiding each other when approached, agonistic interactions did not significantly decrease over time, and solicitations for play or groom were between 10 and 50% successful. In a sequential analysis of captive lesser galagos (*G. senegalensis*), Bercovitch [28] reported that female–female aggression was not systematically followed by an escalation in threats, but most often occurred after one of the females exited a nest box, while Nash and Flinn [29] documented more intrasexual, than intersexual, aggression in the same captive colony upon formation. As opposed to galagos, slow lorises living under the same caged conditions for the same amount of time, with equal 1 male 3 female group formations, spent most of their waking time in proximity to others, often 3–4 animals, agonistic interactions significantly decreased over time, and all forms of play or groom solicitations were 90% successful [30].

Strepsirrhines vary in size and dietary needs, and some are diurnal while others are nocturnal; therefore, their captive housing has to be tailored to each species. Previous housing conditions for pygmy slow lorises included mixed species and enriched enclosures, but due to their endangered status as of 2019, the 50 pygmy slow lorises left in North America are now housed by the Species Survival Plan, and the DLC does not house any lorises of any species [31]. There are earlier reports on captive loris (*Tardigradus nordicus*) housing conditions [32], varying from singly to group housed, while more recent studies on Javan slow lorises (*Nycticebus javanicus*) describe housing them in uni-male multi-female groups [33]. Reports on the housing conditions and social behaviors of pygmy slow lorises under group-housed conditions remain underreported in *Nycticebus*.

As with all primates in captivity, efforts to improve cage space and complexity should be implemented, and previous work has detailed the importance of structures such as branches, nesting, feeding areas, and cage space for captive pygmy slow lorises [34,35]. Pygmy slow lorises belong to the subfamily Lorisinae that cannot leap [36] but have adapted to an arboreal lifestyle as slow climbing specialists [37]. Providing substrates that promote these positional species-specific behaviors should be a captive behavioral and conservation management priority. Pygmy slow lorises are particularly susceptible to stress in nonnatural environments and have the highest infant mortality rates of all prosimian primates in accredited zoos [38]. Positional behaviors are directly correlated to their environmental adaptations and social needs. Bridging and horizontal or vertical suspension from one to all limbs are needed for movements in the canopy, and positional clinging suspension with their hind legs is needed for mating behaviors, while cryptic slow vertical or horizontal postural modes are associated with predator avoidance or feeding behaviors [9,16,37,39]. Previous studies on positional and locomotor behaviors in lorises have been focused on anatomical evolutionary perspectives, substrate preference, and their ecological arboreal adaptations. However, none have focused on a purely quantifiable method for assessing welfare. As improvements in captive conditions include more space and complexity of substrates, their postural and locomotor behaviors should reflect that change.

One method used for assessing the welfare of captive animals is to investigate their activity budgets, especially the time devoted to conspecific interactions [40,41]. Activity budgets of captive pygmy slow lorises have been restricted to singly housed males [42,43], and although, recently, activity budgets of the Bengal slow lorises have been documented in the wild [44], to our knowledge, activity budgets of pygmy slow lorises housed in all-female groups have not been reported. Understanding changes in behaviors and how animals spend their time may be indicative of an improvement of their welfare state. Refining housing conditions by the creation of isosexual groups over solitary housing in primates has been widely used as a behavioral management strategy to reduce stress in captivity [45,46], and more recent work in the formation of male–male pygmy slow loris pairings [5] has been shown to be a viable behavioral management strategy in the reduction in physiological stress while demonstrating their choice to engage in affiliative behaviors and sharing of nesting sites.

Our study examined how the isosexual housing of one all-female pygmy slow loris pair and one multi-female group influenced individual behavior by comparing solitary and group activity. Our goal was to investigate whether or not behavioral changes were indicative of an improvement in captive housing in the female pygmy slow lorises.

The specific objective of this study was to provide new information about the gregariousness of pygmy slow lorises as they adapt to larger enclosures with conspecifics. We predicted that: (1) females in a same-sex group would affiliate with each other, becoming more gregarious, rather than restrict themselves to maintaining a solitary lifestyle; (2) changes in daily activity budgets would occur after the transfer to a more complex environment; and (3) postural and locomotor behaviors would be more varied living in an environment with a variety of climbing structures compared to solitary quarters without more natural substrates.

## 2. Materials and Methods

### 2.1. Study Site

This study was conducted in the Japan Monkey Centre (JMC), Inuyama, Japan. One of the key goals of JMC is to promote the survival and wellbeing of endangered primate species. Between 2006 and 2007, JMC accepted more than 20 pygmy slow lorises that had been confiscated at airports in Japan over the last decade. Given increasing information from the field suggesting that lorises are more gregarious than originally thought, we saw the need to update our husbandry practices to improve the lives of the lorises we housed. Previous reports of socially housing lorises in captivity were reported with both successful, for up to several years, and unsuccessful attempts. Most of these studies were based on solitary or male–female pairs, with no information on isosexual pairings [34,35,47]. To house the confiscated lorises, JMC built the “Slow Loris Conservation Centre” (SLCC) in 2015 to maintain multiple animals in a single unit. The primary goals of SLCC are to enhance the lives of illegally caught lorises by mimicking to the extent possible their natural habitat and to engage in conservation education and science activities [5].

Housing is based on a reverse-lighting cycle, with red-film lights in each room during the night cycle, to minimize light pollution [48,49]. The reverse light–dark cycles have a gradual change over time, with lighting levels at 0.0 to 0.18 lux during the dark phase. The cages were regularly provided with natural branches of various sizes and girths. Wooden nest boxes were provided both in the single and social housing conditions, with branches from natural foliage for hiding places. Enrichment devices such as insect dispensers, wooden devices filled with tree gum, and various feeding stations were placed inside the new larger-enriched enclosures [5]. The diets were mainly composed of natural exudates (gum), various insects, and supplementary vegetables [42,50]. All animals were kept singly housed before the transfer to group housing (see Section 2.2.).

### 2.2. Study Subject Testing Environment

Six adult females brought to the SLCC in 2016 were the focus of this study. All females appeared to be in good health after initial physical examinations but were placed in individual quarantine units as part of standard operating procedures of the JMC. Some animals may have had social interactions with conspecifics for a few years after their confiscation, but it is unclear which individuals had social housing experience and which did not [5].

Females were housed in solitary cages facing each other to promote visual, olfactory, and vocal contact, enabling investigators to monitor subjects for signs of either compatibility or antagonism before pairing, but no signs of aggression or incompatibility were observed. After a period of habituation of several weeks, the cages were placed closer to each other, with enough space to not allow physical contact, in front of their new enriched enclosures for the initial part of the observations in the single caged condition. The quarantine units were steel cages (satellite cages) (610 × 70 × 820 mm; 0.3 m^3^), with a mesh top, bottom, and sides, a wooden nest-box, and a few vertical and diagonal branches. The group formation process was as follows: after an average of 90 days in solitary housing, for the first pair of lorises in the first group, and 150 days for the second group of four lorises, the six females were relocated to a large enriched enclosure containing tree branches, perches, and six wooden nest-boxes. The first group of two females were introduced together into a large enclosure of two separate compartments connected by multiple tunnels (128 cm × 125 cm × 207 cm each compartment) on 25 August 2017, after a few weeks of small one-hour monitored introductions. The group of four individuals were placed into one unit (239 cm × 282 cm × 249 cm) together on 19 December 2017, without small introductions, as the largest enclosure was not constructed, to allow for the quick emergency separations of one individual from others. All observations in the post-social-housing conditions were made once the groups were formed without monitored separations. The potentially aggressive activities that we observed occurred in the first few introduction trials for the one pair of females. They engaged in minor hair clasps and leg grabs, and it was unclear if the initial chasing bouts were antagonistic or playful. The group of 4 also had a few events of clasping, chasing, and one bite not requiring veterinary intervention on the introduction day, but none were seen after that and they all shared the same nest from day 2. As we started our formal observations once the groups were established, and no aggression occurred during our observations, we did not report it in our findings. The period after the introduction is referred to as the social housing condition. 

### 2.3. Data Collection

Behavioral data were collected in 10 min sampling sessions following Martin and Bateson [39] and are described in Table 1. Postural data classification followed Glassman and Wells [25] and is described in Table 2.

Daily 10 min focal recording sessions for each female were made with a night vision video camera during the night-cycle between 13:00 and 16:00 from June 2017 to March 2018, with an average of 3.14 (± 1.21; standard deviation SD) observations per individual weekly. The same methods were used before and after the group formation. Observations for the pair group before the social housing started were conducted from June through September (3 months) and from September to February (5 months) post-social-housing, while for the group of 4, pre-social-housing was performed from November through December (2 months) and December through February (3 months) in the social-housing condition. We conducted instantaneous sampling [51] at one-minute intervals during the 10 min focal sample. Social gregariousness or solitariness was assessed based upon proximity. We divided proximity scores into three categories: far (opposite side of enclosure), close (within 2 m), and social (arms-reach). Positional behaviors were divided into 4 groups for statistical purposes (see Table 2). We recorded behavior using the night-mode video cameras. We collected a total of 2740 min of behavioral data, with an average of 30 min per week (solitary: ± 56.21; SD-social: ± 118.77; SD) of data per subject.

### 2.4. Statistical Analyses

We used the Kruskal–Wallis rank sum test to evaluate the effect of housing condition on proximity, and then performed a post hoc Dunn test with Bonferroni correction. We used analysis of variance (ANOVA) to test for differences in (1) activity and (2) positional behavior between different periods. “Activity” was used as the dependent variable and “period” as the independent variable. Where main effects were significant, we used the post hoc Tukey’s HSD test for pair-wise comparisons. All datasets were checked for normality with the Kolmogorov–Smirnov test and for homogeneity of variance using Levene’s test (*p* > 0.05). Statistical significance was set at *p* < 0.05 and two-tailed results were reported. *P*-adjusted values were used for multi-pair comparisons where ANOVA was conducted with nonhomogeneous data. All statistical analyses were conducted with RStudio version 4.1.0 (R Foundation for Statistical Computing, Vienna, Austria).

## 3. Results

### 3.1. Proximity

**Figure 1:**
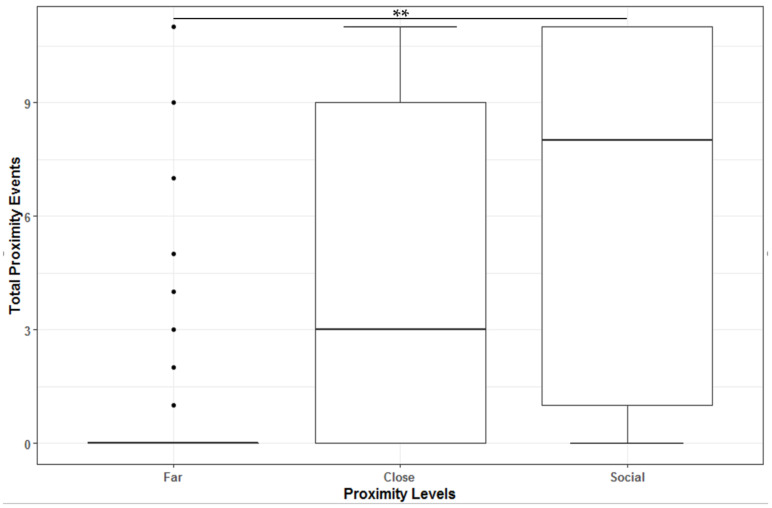
Boxplot representing all observations in the three types of proximity, where the boxes represent the lower and upper quartiles, whiskers represent the minimum and maximum observations, and horizonal line represents the median, with significant codes set as: ** 0.01.

Female pygmy slow lorises displayed a high degree of gregariousness when transferred to the group condition (Kruskal–Wallis chi-squared = 10.71, df = 2, *p* = 0.0047). Post hoc tests showed that pygmy slow lorises were significantly more likely to spend their time at proximity under a social condition inside their nesting sites (Far – Social: z = −3.14, *p* = 0.0051). There were no significant differences between Close – Far (z = 2.38, *p* = 0.052; and between Close – Social (z = −0.76, *p* = 1.0). They spent on average around 60% of their time at arm’s reach (close) (Figure 1).

### 3.2. Activity Budgets

**Figure 2:**
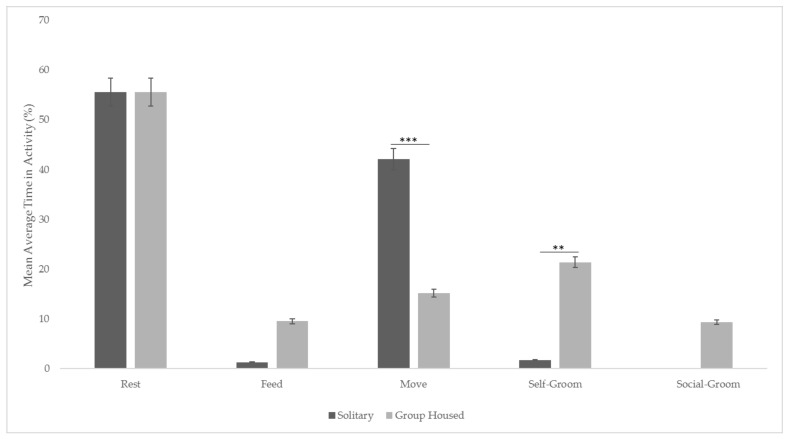
Activity budgets before and after group formations with bars representing standard error SE and Significant. Codes set as: *** 0.001 ** 0.01.

There was a statistically significant difference between activities in different periods (F (1,1240) = 7.059; *p* = 0.007). Post hoc tests showed that they spent significantly less time moving (*p* < 0.001) and more time self-grooming (*p* = 0.002) when under the group condition, but there were no significant changes in resting (*p* = 0.631) or feeding time (*p* = 0.973) between the two housing conditions. Social grooming could not be compared between the two housing conditions, but when housed with conspecifics, female pygmy slow lorises spent an average of 21.3% self-grooming and 9.36% of the time grooming each other (Figure 2).

### 3.3. Positional Behaviors

**Figure 3:**
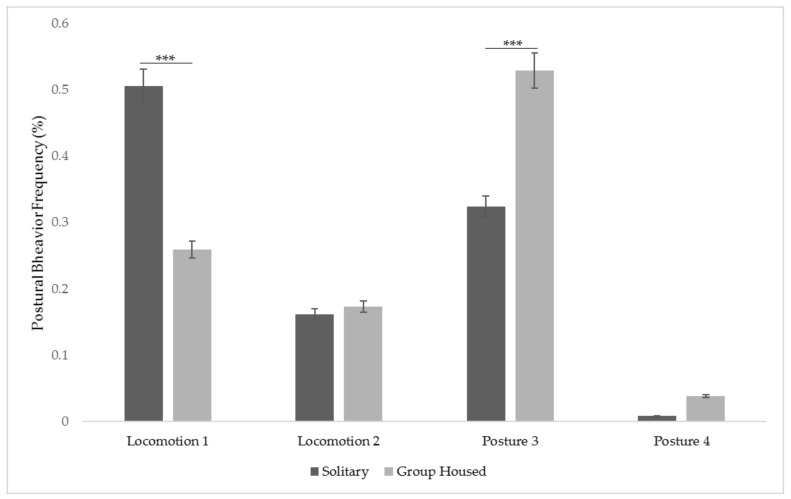
Locomotor postures and postural modes before and after group formations with bars representing standard error SE and significant codes set as: *** 0.001.

There were no significant differences between postural behaviors under different housing conditions (solitary vs. social housing) (F (3,3020) = 0.02; *p* = 0.88). Post hoc tests showed that there was a significant decrease in Locomotion/Posture 1(*p* < 0.001) and a significant increase in Locomotion/Posture 3 (*p* < 0.001), but no significant differences in Locomotion/Posture 2 (*p* = 0.89) or in Locomotion/Posture 4 (*p* = 0.98) (Figure 3).

## 4. Discussion

### 4.1. Sociality among Female Pygmy Slow Lorises

The group housing of adult male pygmy slow lorises as a behavioral management strategy has been shown to improve their well-being [5]. As with their male counterparts in the previous study [5], the newly formed female social groups showed little to no aggression, even though twice as many animals (4) were introduced into the same amount of space per group. Our study provides further evidence that pygmy slow lorises are not only gregarious animals, but also not antagonistic toward conspecifics. Female–female dyads were quicker to form affiliative relationships when compared to our male–male pairs [5], providing a stronger case for female pairings as a viable behavioral management strategy to improve loris well-being in captivity. Dyads slept together in the same wooden nest-boxes, rather than alone, and they spent about 10% of their time grooming each other.

It has been shown that lorises in captivity (*Nycticebus coucang*) prefer to sleep in dark areas of their enclosures, especially in wooden nest-boxes when natural foliage is not readily available [52], and our findings show that females preferred affiliative interactions in the nesting area over more ample and solitary spaces. At the SLCC, all groupings of males and females interacted in similar ways when housed with same-sex conspecifics. Both sexes tended to sleep with others, not alone, and both readily engaged in allogrooming. However, one difference is that females, unlike males, did not change nesting sites after the first day of introduction, while males slept in different nests over time [5]. This resembles findings in wild populations of slow pygmy lorises in Cambodia, where females returned to the same sleeping sites while males did not [17]. In captivity, *Nycticebus coucang* females housed together have been reported to actively seek each other for grooming and affiliative interactions [30], and our females also behaved similarly. Our findings also resemble those found in female macaques housed in laboratories [53]; when continuously paired over intermittently paired, females show a high degree of affiliation. It may not be possible to provide social housing to all animals, but whenever possible, all-female social housing should be tried in slow pygmy lorises over single housing to improve their captive care.

### 4.2. Activity Budget

Our findings supported our predictions that daily behavioral activity changed once the female pygmy slow lorises moved to their socially enriched enclosure, with a decrease in movement and an increase in grooming. Social grooming has been reported to help maintain group cohesion, reduce intergroup hostility [54], assist in establishing and maintaining dominant–subordinate relations, and informing nonkin alliances [55,56]. The female pygmy slow lorises in our study started allogrooming after group formations, suggesting that they are a relatively gregarious species. One would not expect solitary species to interact in this manner. For example, koalas (*Phascolarctos cinereus*) are a solitary species, and when females are housed together, they rarely, if ever, sit in the same tree (Bercovitch, pers. obs.). On the other hand, self-grooming significantly increased among female pygmy slow lorises housed together and is often considered a sign of increased stress [57]. However, self-grooming is context-dependent, and for *Nycticebus* spp., self-grooming plays important and complex roles. Pygmy slow lorises rely heavily upon olfactory cues for mate selection, competition, and territorial behaviors. Lorises use the chemical compounds in their saliva mixed with secretion from their brachial glands as a protection from predators, as well as ectoparasites, and their glands become active as young as 6 weeks old [58]. In the wild, self-grooming may function to reduce ectoparasites in various primates. Fisher et al. [19] studied countermarking among singly housed captive pygmy slow lorises at the San Diego Zoo and demonstrated the importance of chemical signaling in their behavioral repertoire. It has been hypothesized from recent findings that lorises undergoing solitary torpor or infant parking may self-groom, covering themselves or their infants with protective venomous saliva [10]. This appears to be a naturally occurring behavior that may not necessarily indicate stress or poor welfare in captivity. Ehlrich and Musicant [30] reported that Sunda slow lorises (*Nycticebus coucang*), living socially in one-male-multi-female groups of wild-caught individuals in captivity, had grooming and self-grooming at the top of their activities in social behaviors, with 5% of their time spent in social grooming and 8% in self-grooming under social conditions. Grooming was also a common behavior noted during bouts of play. In the same species (*Nycticebus coucang*), some studies have reported that males self-groomed more than females [58,59], while other studies [60] have documented the opposite trend. In summary, while allogrooming is an indicator of sociality, self-grooming need not be an indicator of elevated stress and could even be adaptive for pygmy slow loris chemical communication.

Translocation to the larger social enclosure resulted in a significant decrease in moving time, which seemed to be compensated for by increases in both self and social grooming. Although not statistically significant, the increased time spent eating could have resulted from access to enrichment devices with insects and tree gum that were not available in their small cages, due to a lack of space. We suggest that the larger enriched enclosures with feeding devices such as tree logs with gum may have provided more opportunities for greater diversity in food resources, and more natural feeding postures [61], which should enhance animal welfare. Given the high level of sociality that appeared when placed with conspecifics, the principal finding of our study is that female pygmy slow lorises probably benefit from living in an enriched large enclosure with other females because it provides an avenue to express their natural behaviors. As reported by Yamanashi et al. [5], male lorises opted to feed, travel, and nest together, suggesting that both sexes are more gregarious than often assumed. In the wild, both male and female pygmy slow lorises have been found to have a large home range relative to their small body size [17]. The pygmy slow lorises engaged in social behaviors such as feeding together, as well as social grooming. After an initial exploration of their new enclosure, time was most likely better spent in social behaviors rather than locomotion. The complexity of the social-housing condition of both enclosures provided more behavioral opportunities. Leisure time, social grooming, and resting with other females in the wooden nest-box seem like plausible explanations for a significant decrease in moving time.

### 4.3. Postural and Locomotor Behaviors

Although there were some differences in the setup of the pair and four group enclosures, both had access to a dozen climbing structures, vines, perching, platforms, and other structures where they could suspend themselves from. In general, female pygmy slow lorises decreased the time spent moving when in the larger enclosure, as well as increasing the times spent stationary. We suggest that this combination of findings mimics the natural postural and locomotor behavior of the species in their natural habitat. Our findings resemble those reported for socially housed slow lorises (*Nycticebus coucang*) that spent more time sitting as the most important postural behavior in social interactions when in proximity to several animals, and our pygmy slow lorises behaved the same while self-grooming and allogrooming [30]. In our study, a likely possibility is that sitting increased because the animals were grooming more, and thus spending less time in quadrupedal locomotion. The lack of significant differences in other postures between solo and social housing could have resulted from the relatively enhanced conditions of the solo enclosures, where animals could still cling to branches and move about on multiple substrates. The larger and more complex social enclosure was essentially a larger, and more complex, scale of the heavily enriched small solitary cages. Although not significant, one postural mode behavioral difference that we did record was in “bridge” behavior. Instead of jumping or leaping from tree to tree, a common postural accommodation for moving across various substrates among lorises is “bridge” [39]. This activity is used by an individual to pull a branch toward it in the canopy and elongate his body to travel across the canopy from branch to branch without having to go down to the ground, exposing itself to predation. The enriched small enclosure might not have had enough room above the horizontal bridge for them to express this behavior as much. Contrastingly, the significant decrease in climbing up or down, or walking on all fours around the enclosure, was most likely due to their increase in time inside the one wooden nest-box shared by all. A decrease in some locomotive postures may be indicative of a decrease in stress-related behaviors such as pacing (one female engaged in stereotypic pacing before being socially housed), while an increase in postures such as sitting may be related to more relaxed states and affiliative interactions that could be associated with compatibility with cage mates and an increase in affiliative interactions. A behavioral management plan that includes scanning postures of an animal that is hard to observe because of their size and lighting conditions could give us insight into their welfare state.

## 5. Conclusions

Our findings show that the formation of all-female groups in pygmy slow lorises is a feasible behavioral management strategy that promotes natural behaviors. A high degree of social behaviors displayed in the group environment challenges early reports of them being highly solitary in the wild. Daily activity budgets can change once placed under an enriched social housing condition. Both substrate complexity and enclosure size seem to mediate what postural behaviors are adopted. We conclude that pygmy slow lorises are a gregarious species that can benefit in captivity if housed more often with conspecifics of both the opposite and same sex, in large enclosures with complex substrates that also allow for a more naturalistic postural behavior repertoire and diet.

## Figures and Tables

**Table 1:** Behavioral ethogram. Based on Nekaris [9] and adapted to our study.

Behaviors	Description
Rest	The body is immobile and not engaging in any activity
Feed	Consuming any animal or provisioned food or mastication
Move	Any mobile activity in any direction
Self-Groom	Licking or combing with teeth its coat
Social-Groom	Actively licking or combing with teeth a conspecific’s coat or receiving the same

**Table 2:** Postural modes and locomotor postures. Postural behaviors adapted from Glassman and Wells [39].

Postural Categories	Posture	Description
Locomotion Posture 1	Climb up	climbing up using all limbs
	Climb down	climbing down using all limbs
	Quadrupedal walk Cling	walking on all four limbs hanging with more than two limbs
Locomotion Posture 2	Quadrupedal stand	standing with all four legs
	Bipedal stand Bipedal hang	standing with hind legs hanging face down with hind legs
Positional Posture 3	Sit Sleep ball	sitting or lying down curled up in a ball
Positional Posture 4	Bridge	extending all limbs to grab or move between two substrates
	Other	unclear posture or transitional position

## Data Availability

The data presented in this study are openly available in FigShare at doi:10.6084/m9.figshare.16553079.
